# Ocular Manifestations in Patients with Werner Syndrome

**DOI:** 10.3390/ijms27073187

**Published:** 2026-03-31

**Authors:** Toshiyuki Oshitari, Masaya Yamaga, Yoshiro Maezawa

**Affiliations:** 1 Department of Ophthalmology and Visual Science, Chiba University Graduate School of Medicine, Inohana 1-8-1, Chuo-ku, Chiba 260-8670, Chiba, Japan; 2 Department of Ophthalmology, International University of Health and Welfare School of Medicine, Kozunomori 4-3, Narita 286-8686, Chiba, Japan; 3 Department of Diabetes, Metabolism and Endocrinology, International University of Health and Welfare Narita Hospital, Hatakeda 852, Narita 286-8520, Chiba, Japan; 4 Department of Endocrinology, Hematology and Gerontology, Chiba University Graduate School of Medicine, Inohana 1-8-1, Chuo-ku, Chiba 260-8670, Chiba, Japan

**Keywords:** Werner syndrome, juvenile cataracts, *WRN*, optical coherence tomography, cystoid macular edema, glaucoma, pachychoroid, delayed wound healing

## Abstract

Werner syndrome is a rare autosomal recessive premature aging syndrome characterized by its development after puberty and death in patients in their 50s due to cancer or atherosclerotic disease. Early diagnosis can improve the management of disease, quality of life and prolong the lifespan of patients with Werner syndrome. Ophthalmologists should include Werner syndrome in the general work-up in patients with bilateral early-onset cataracts. We present a case of Werner syndrome with initial signs of juvenile cataracts. The patient had a high-pitched voice, a bird-like face and progeroid hair. We performed routine ophthalmological examinations including slit-lamp examinations, fundus examinations, and optical coherence tomography, and genetic analysis. The patient had plateau iris and pachychoroid-like features in addition to bilateral cataracts. The gene analysis revealed compound heterozygosity of *Mut4* and *Mut25* in *WRN* and the patient was diagnosed with Werner syndrome. After cataract surgeries, his visual acuities were improved. Additionally, we performed a thorough literature review to better understand the previously reported ocular manifestations in patients with Werner syndrome.

## 1. Introduction

Werner syndrome is a rare autosomal recessive inherited disorder caused by pathogenic variants in the *WRN* gene on chromosome *8q12*, which encodes a RecQ-type DNA helicase [1]. The clinical manifestations of this premature aging syndrome include gray hair, hair loss, bilateral cataracts, skin changes, soft tissue calcification, a bird-like appearance, abnormal voice, diabetes, and osteoporosis [2]. The incidence of Werner syndrome is one in 20,000–40,000 in Japan, which is ten times higher than that in the United States of America [3]. The incidence of Werner syndrome in the world is one in 1,000,000–10,000,000 and, thus, there are approximately 1400 patients with Werner syndrome in the world [4,5]. The average lifetime is in the 50s, and the causes of death are malignant tumors and cardiovascular diseases [5,6,7]. The Japanese Werner Consortium has been working on various contributions for decades, including the development of diagnostic criteria and the establishment of patient registration [2,8,9]. Owing to long-term efforts, the life expectancy of patients with Werner syndrome has been extended: the average lifespan between 1997 and 2006 was 51.8 years [2], the average age between 2006 and 2008 was 55.0 years [5], and the recent mean age between 2011 and 2020 was 59.0 years [10]. The most common cause of death was malignant tumors, but no patient died due to cardiovascular events in a recent survey [10]. Therefore, we propose that early diagnosis enables early intervention and better management of the patients’ health, notably in the case of cardiovascular disease.

Bilateral cataracts are one of the major and early cardinal signs in patients affected by Werner syndrome, and a recent report using the registry indicated that cataracts are always present [11]. A recent survey suggested that in young patients with Werner syndrome, bilateral cataracts, hair changes, and low body weight are the most frequent signs. Furthermore, the research group reported that, in addition to bilateral cataracts and hair changes, the presence of one of the following gives a suspicion of Werner syndrome in patients aged less than 30 years: short status, abnormal glucose or lipid metabolism, and skin atrophy [12]. Due to the fact that bilateral early-onset cataracts are always present, ophthalmologists play a significant role in the early diagnosis of Werner syndrome in patients in their 20s because early diagnosis leads to an improved lifespan and quality of life.

Although bilateral cataracts are important cardinal signs and symptoms in patients with Werner syndrome, other ocular manifestations such as glaucoma, corneal opacity, or retinal and choroidal abnormalities have been reported.

The *WRN* protein has both DNA helicase and endonuclease activities and is known to play a role in DNA replication, gene transcription, telomere maintenance, and DNA damage repair [13,14]. Although dysfunction of *WRN* is involved in aging, the precise mechanisms of action of *WRN* in ocular manifestations remain unclear. Here, we present a case of Werner syndrome and summarize the ocular manifestations that accompany Werner syndrome. Furthermore, we discuss the potential role of *WRN* in the development of ocular manifestations.

## 2. Case Report

### 2.1. Case Presentation

**Figure 1:**
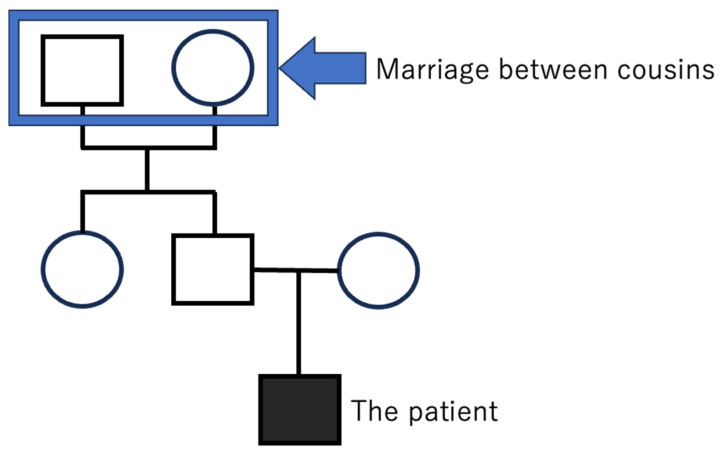
A simple figure with the pedigree of the patient. His father’s parents were married between cousins. His father had diabetes mellitus and malignant lymphoma. Squares are male. Circles are female.

A 26-year-old man noticed a decrease in vision in both eyes at the age of 24 years. He was diagnosed with bilateral cataracts at a former clinic one year ago and was referred to our hospital for further examination and therapy. His father’s parents were married between cousins and thus the patient originates, in part, from a consanguineous family (Figure 1).

At the first visit, his best-corrected visual acuities (BCVAs) measured on the Snellen scales were 0.05 × S + 8.0D in the right eye (OD) and 0.7 × S + 3.75 Cyl-1.5D Ax 70 in the left eye (OS). The intraocular pressure was 19 mmHg in both eyes (OU). The axial lengths were 21.6 mm (OD) and 22.2 mm (OS). Fundus examinations were almost undetectable on the right side but normal on the left side. The patient had a high-pitched voice.

**Figure 2:**
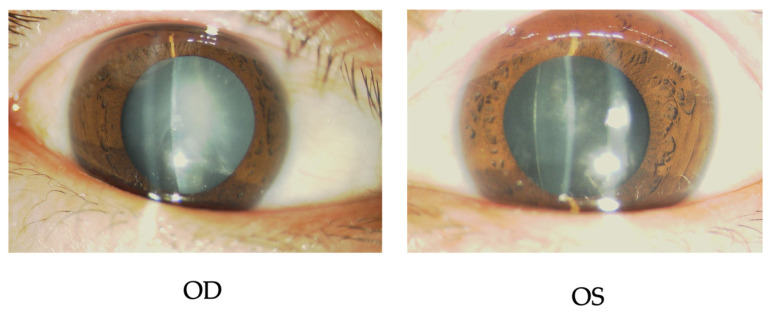
The slit-lamp examinations at the first visit. Bilateral whitish cataracts are observed. OD, oculus dexter (right eye); OS, oculus sinister (left eye).

Slit-lamp examination revealed bilateral cortical and posterior subcapsular cataracts (Figure 2). The right lens was almost milky and whitish. The angles were relatively narrow in grade 2 according to Scheffer’s classification and a plateau iris was observed in both eyes.

Because he had bilateral cataracts and a high-pitched voice, and was suspected of having Werner syndrome, the patient was referred to the Department of Diabetes, Metabolism, and Endocrinology of our hospital for general and metabolic work-up for bilateral cataracts of early onset. Further examinations in the Department of Diabetes, Metabolism, and Endocrinology revealed progeroid hair, an abnormal voice, and a relative bird-like face with cardinal signs and symptoms, a short stature (162 cm), and low body weight (49 kg). No other signs, including skin changes, short tissue calcification, abnormal glucose and/or lipid metabolism, deformation and abnormality of the bone, malignant tumors, premature atherosclerosis, or hypogonadism were identified. He was clinically diagnosed with Werner syndrome and underwent genetic examinations for *WRN* gene pathogenic variants at Chiba University after obtaining informed consent. Genetic analysis was performed at the Kazusa DNA Research Institute using targeted next-generation sequencing (NGS) with a hybrid capture method. The genetic analysis revealed compound heterozygosity of *Mut4 (3139-1G>C)* and *Mut25 (3244delG)* in *WRN*. A final diagnosis of Werner syndrome was made.

### 2.2. Ocular Management of the Patient

**Figure 3:**
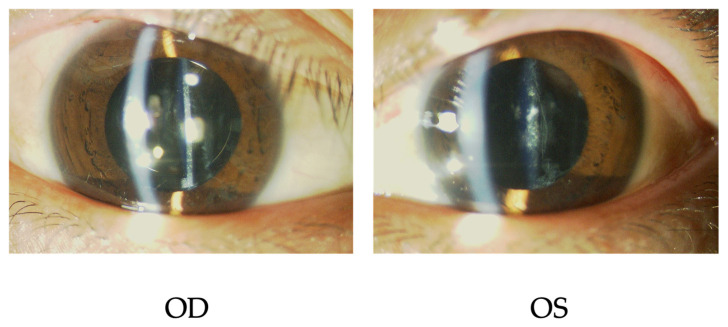
The slit-lamp examinations after the bilateral cataract surgeries. Subcapsular opacities cannot be removed completely but the BCVAs were improved to 1.2 (OU). The patient did not make any complaints about his vision. OD, oculus dexter (right eye); OS, oculus sinister (left eye).

Three months later, phacoemulsification, aspiration, and intraocular lens implantation (PEA + IOL) were combined with goniosynechialysis in the right eye and PEA + IOL in the left eye without complications. Surgical wounds were sutured using 10-0 nylon. His BCVAs improved to 1.0 (OU) two weeks after the operation, and the subcapsular opacities could not be removed completely (Figure 3).

**Figure 4:**
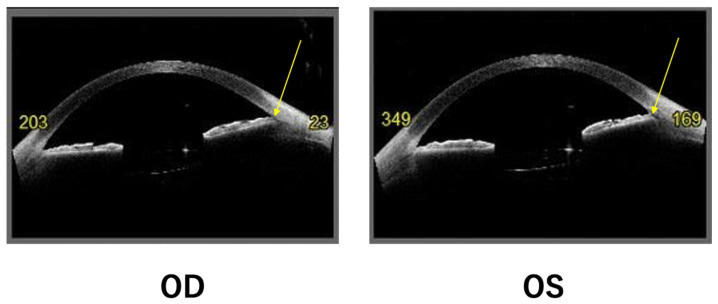
Postoperative anterior segment optical coherence tomography findings. The plateau iris is left (arrows) which may cause postoperative high intraocular pressures in both eyes. OD, oculus dexter (right eye); OS, oculus sinister (left eye).

**Figure 5:**
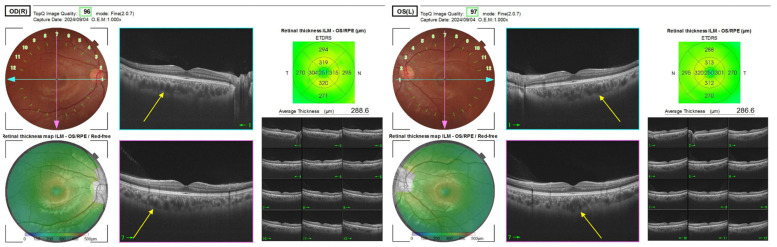
Posterior segment optical coherence tomography findings postoperatively. Vasodilation in the Haller’s layer is observed in both eyes (arrows). These findings suggest the eyes had the pachychoroid-like features.

Because a plateau iris was found, goniosynechialysis was performed in the right eye, but the intraocular pressure increased to 34 mmHg (OD) after the surgery. Therefore, goniosynechialysis was not performed in the left eye. The intraocular pressure was 27 mmHg (OS) after surgery, which was controlled by anti-glaucoma eye drops and finally decreased to 18 mmHg (OS) with carteolol hydrochloride eye drops. Postoperative anterior segment optical coherence tomography showed that the plateau iris remained (Figure 4), which may have caused the increased postoperative intraocular pressures in both eyes. Postoperative fundus examinations were normal, but posterior segment optical coherence tomography showed a thickening of the choroidal membrane due to vasodilation in the Haller’s layer. These findings suggested that the eyes had pachychoroid-like features (Figure 5). Cystoid macular edema was not observed in either eye after surgery. Although high intraocular pressure was temporarily observed after the surgeries, no large cuppings were observed and Humphrey visual field tests were normal.

Eight months after the surgeries, the BCVAs were 1.2 (OU) and the intraocular pressure was 18 mmHg (OU) with carteolol hydrochloride eye drops. The patient was under observation at our hospital because of persistent posterior capsular opacities and increased intraocular pressure. Informed consent was obtained from the patient for the presentation of his clinical findings.

Summaries of ophthalmological findings and procedures are displayed in Table 1.

## 3. Discussion

The patient had a plateaued iris and pachychoroid-like features. This is the first report of a patient with Werner syndrome with a plateau iris and pachychoroid-like features. The patient had a small eye, with axial lengths of 21.6 mm (OD) and 22.2 mm (OS). The bases of the plateau iris are thought to be tightly adhered to the angle and are not detached after surgery, which results in increased postoperative intraocular pressure. In addition, hyperopic eyes tend to have thicker anterior sclera, which may increase the outflow resistance of the vortex vein [15,16]. Short axial length is an intrinsic risk factor of central serous chorioretinopathy [15,16,17]. Choroidal venous congestion caused by an increase in the outflow resistance of the vortex vein can lead to the development of pachychoroidal vessels [18]. Thus, the short axial length was thought to have caused pachychoroidal vessel formation in this case. We previously reported that thinning of the choroid is observed in patients with Werner syndrome because choroidal thickness decreases with age [19]. However, in these three cases, the axial lengths were 23.68/23.60 mm, 24.83/24.82 mm, and 24.52/24.49 mm, respectively, which were longer than this case [19]. Thus, pachychoroidal changes were not observed in these three cases [19].

Our patient had bilateral cataracts and other clinical signs, such as a high-pitched voice; therefore, we suspected the possibility of Werner syndrome early on. Due to the high incidence of bilateral cataracts, ophthalmologists play an important role in the diagnosis of Werner syndrome in patients with early-onset cataracts. When ophthalmologists encounter bilateral cataracts at a younger age, they should be observant for other systemic features; whether present or not, patients with early-onset bilateral cataracts require referral for systemic/general medical work-up. Early diagnosis in patients in their 20s can lead to an improved lifespan and quality of life in patients with Werner syndrome.

Manifestations other than bilateral cataracts have also been reported in patients with Werner syndrome. Therefore, further studies are needed to investigate the changes in the microstructures of ocular tissues in Werner syndrome. Previously published ocular manifestations including ours are displayed in Table 2.

## 4. Review of Ocular Manifestations of Werner Syndrome

### 4.1. Bilateral Cataracts

Bilateral cataracts are one of the most common cardinal signs and symptoms in patients with Werner syndrome, with a prevalence of 100% in each eye and 96% in both eyes [2,11]. In the patients’ 20s, the prevalence of bilateral cataracts is over 90% [12]; thus, detecting bilateral cataracts is essential for the early diagnosis of Werner syndrome in younger patients [12]. A variety of cataract characteristics are observed in patients with Werner syndrome, including subcapsular posterior cataracts [20], hypermature or Morgagnian cataracts [21], milky and opaque cataracts [22], and nuclear sclerotic cataracts [23]. These characteristics cannot be differentiated from other causes of cataracts, such as drug-induced cataracts, injury, infection, or inflammation. However, if bilateral nuclear sclerotic cataracts are found in individuals in their 20s or 30s, ophthalmologists should suspect Werner syndrome because nuclear sclerotic changes may be related to aging [23]. The progression of cataracts in patients with Werner syndrome seems to be rapid; thus, patients may initially consult ophthalmologists because a decrease in vision is the first clinical sign.

The precise mechanisms underlying the development of bilateral cataracts in patients with Werner syndrome are still unknown. However, Zhu et al. examined promoter methylation and histone modification of *WRN*, and changes in *WRN* expression in the age-related cataract lens [36]. Their study suggests that WRN protein expression in the anterior lens capsules in age-related cataracts is lower than that in the control, and that the methylation status in region 3 of the *WRN* promoter in age-related cataracts is significantly higher than that in the control [36]. Furthermore, hypermethylation of H3-K9 has been observed in age-related cataracts [36]. The precise mechanisms underlying age-related cataract development remain unclear; however, oxidative stress and excessive production of reactive oxygen species are associated with the development and progression of age-related cataracts [37]. *WRN* is associated with double-strand break repair pathways and is required for cellular DNA replication and repair [38]. A decrease in *WRN* expression in the anterior lens capsules in age-related cataracts indicates a reduction in DNA repair capability, followed by the inadequate repair of oxidative DNA damage in age-related cataracts [36]. Thus, aberrant epigenetic methylation of the *WRN* gene and the associated histones may be related to the reduction in *WRN* expression and the development of age-related cataracts [36]. Similarly, *WRN* gene pathogenic variants, followed by WRN protein dysfunction, may be associated with early lens opacity in patients with Werner syndrome.

Various complications after cataract surgery in patients with Werner syndrome have been reported, but most of them occur after extra- and/or intracapsular cataract extraction with a large incision [20,31]. Thus, these complications are thought to be related to wound dehiscence; that is, peripheral anterior synechia, secondary epiretinal gliosis, cystoid macular edema, unplanned filtering blebs, and corneal endothelium decompensation [20,31]. Wound dehiscence followed by spontaneous bleb formation can occur ten years after surgery [32]. However, the mechanisms underlying tissue repair and regeneration impairments remain unclear. However, Tu et al. indicated that in a bone defect model and a cutaneous wound model of mice, hepatocyte growth factor (HGF) was downregulated in *WRN*^−/−^ mesenchymal stem cells, resulting in poor angiogenesis and cutaneous wound healing [39]. Because angiogenesis and wound healing induced by HGF are regulated by the phosphatidylinositol-3 kinase (PI3K)/Akt pathway, HGF insufficiency and PI3K/Akt dysregulation impair the pro-angiogenic function of *WRN*^−/−^ mesenchymal stem cells [39]. These results indicated that trophic disruption between stromal and epithelial cells may be associated with impaired wound healing in patients with Werner syndrome [39].

The impairment of wound healing was resolved by a small incision surgery, PEA + IOL [22,33]. Our patient had no severe complications during or after surgery in either eye. However, even if the small incision surgery is selected, cystoid macular edema can develop after the surgery [23,33,34]. Once cystoid macular edema develops, some cases become intractable [23,34]. The mechanisms underlying the development of cystoid macular edema after cataract surgery are also unknown; however, we have shown that *WRN* is expressed in the Müller cells of healthy human retinas [34]. Because the WRN protein is expressed in the cytosol of Müller cells, WRN protein may have other roles as a DNA helicase or exonuclease in the cytosol. For example, WRN can interact with p53, activate the p53-dependent transcription of p21/Waf1, and may be involved in apoptosis [40,41]. Because pathological changes in Müller cells are known to be associated with the development of cystoid macular edema [42,43,44], the absence and/or dysfunction of the WRN protein in Müller cells in patients with Werner syndrome may be related to the development of cystoid macular edema associated with Müller cell dysfunction [34].

### 4.2. Glaucoma

Glaucoma is an age-related ocular disease accompanied by retinal ganglion cell death and retinal nerve fiber loss, and its prevalence increases with age [45]. Aging is a major risk factor for the development of glaucoma [46]. The thickness of the retinal nerve fiber layer measured using optical coherence tomography is useful for the early diagnosis of glaucoma [47]. A recent cohort indicates that in patients with well-controlled glaucoma, the average rate of change in the retinal nerve fiber layer thickness is −0.70 μm/year and older patients have faster rates of retinal fiber layer loss than younger patients at the same level of intraocular pressure [48]. On the other hand, the thickness of retinal nerve fiber layer is known to decrease with age by −0.3 μm/year in healthy individuals [49].

Werner syndrome is a premature aging disorder; thus, the thickness of the retinal nerve fiber layer is expected to be lower than that in age-matched controls. We have reported several cases of Werner syndrome with a thinner retinal nerve fiber layer and glaucomatous optic neuropathy [19,24]. Abraham et al. reported three cases of Werner syndrome accompanied by primary glaucoma [25]. They postulated that connective tissue abnormalities in the trabecular meshwork and compromised vascular supply surrounding the optic nerve head may cause glaucoma development in patients with Werner syndrome [25]. The accumulation of oxidative mitochondrial DNA damage may be related to age-associated neurodegenerative diseases such as Alzheimer’s disease, Parkinson’s disease, and Werner syndrome [50]. The ganglion cells in Werner syndrome may be susceptible to age-related oxidative stress. Further studies are required to elucidate the association between neurodegeneration and *WRN* pathogenic variants. The prevalence of glaucoma in patients with Werner syndrome is unclear because of the small sample size; however, ophthalmologists should monitor glaucoma in addition to cataract surgery in patients with Werner syndrome.

### 4.3. Corneal Diseases

Corneal endothelial decompensation is one of the major complications after classical cataract surgeries in patients with Werner syndrome [20,31]. However, small incision cataract surgeries do not significantly affect the corneal endothelial cells. Kemmanu et al. performed endothelial cell counts before and after PEA + IOL and YAG capsulotomy in a patient with Werner syndrome [35]. This study indicated that endothelial cell counts decreased by 8.6% after 15 months of PEA + IOL and 11 months of YAG capsulotomy, which is within the acceptable range of cell loss after surgical procedures [35]. Thus, modern surgical procedures for cataracts in patients with Werner syndrome are safe for the corneal endothelial cells. Singh et al. recently reported primary bullous keratopathy in a patient with Werner syndrome [26]. As bullous keratopathy develops spontaneously, premature aging can be attributed to the progressive loss of corneal endothelial function [26]. Kremer et al. reported bilateral corneal metastatic calcification in two patients with Werner syndrome [27]. In this study, histopathological examinations supported metastatic corneal calcification but not calcific band keratopathy [27]. Ectopic soft tissue calcification is a well-known symptom of Werner syndrome, and both calcification and Pit-1 overexpression are detected in the skin of patients with Werner syndrome in situ [51]. Thus, overexpression of Pit-1 may be involved in the formation of soft tissue calcification [51] and corneal stromal calcification in two cases of Werner syndrome [27].

### 4.4. Other Ocular Manifestations

Other ocular manifestations are extremely rare; however, retinitis pigmentosa-like features [28], paramacular degeneration [29], and bilateral retinal detachment [30] are very rare ocular manifestations of Werner syndrome. In one case of bilateral retinal detachment, chorioretinal atrophy and excessive vitreous liquefaction were observed; vitreoretinal changes were found to be involved in premature aging in Werner syndrome [30]. WRN protein expression is found in human retinas [34]; therefore, *WRN* gene pathogenic variants may be, in part, involved in retinal abnormalities in these patients [28,29,30].

## 5. Materials and Methods

The clinical study was done in accordance with the Declaration of Helsinki and consent was obtained. The study was approved by the Institutional Review Board of Chiba University Hospital (protocol code HS202409-01 and approval date: 1 September 2024) and written informed consent has been obtained from the patient to publish this paper.

### 5.1. Clinical Examinations and Surgical Procedures

For case presentation, routine ophthalmological examinations, slit-lamp examinations (700GL, TAKAGI SEIKO, Tokyo, Japan), fundus examinations, measurements of intraocular pressures (NT-530, NIDEK, Gamagori, Japan), Humphrey visual filed tests (HVF III840, ZEISS, Oberkochen, German), and anterior (CASIA II, TOMEY, Nagoya, Japan) and posterior OCT (DRI OCT Triton, TOPCON, Tokyo, Japan) examinations were performed. Measurements of visual acuity were done and the results were expressed on the Snellen scales. Other examinations performed were specular microscopy (CELLCHECK20-P, KONAN, Nishinomiya, Japan), IOL master (IOL master model 700, ZEISS, Oberkochen, German), and autofluorescence angiography (Optos Panoramic Ophthalmoscope California, Nikon, Tokyo, Japan). Cataract surgeries were performed using the small incision technique, PEA + IOL in both eyes, and goniosynechialysis was combined in the right eye. The incision was performed at the corneoscleral limbus and surgical wounds were sutured using 10-0 nylon.

### 5.2. Genetic Analysis

Genetic analysis was performed at the Kazusa DNA Research Institute using targeted next-generation sequencing (NGS) with a hybrid capture method, focusing on the exonic and flanking intronic regions of *WRN*, *LMNA*, *BLM*, *RECQL4*, *RMI1*, and *POLD1*.

## 6. Conclusions

Bilateral cataracts are a major cardinal sign of Werner syndrome and are frequently observed in younger patients. Because early diagnosis and intervention can improve the lifespan and quality of life of patients with Werner syndrome, ophthalmologists should consider Werner syndrome as one of the causes of bilateral juvenile cataracts. At the same time, other ocular manifestations such as glaucoma, corneal diseases or retinal abnormalities can accompany Werner syndrome. Modern cataract surgeries are safe for patients with Werner syndrome. However, surgeons should be aware of the development of cystoid macular edema after cataract surgery. The precise mechanisms underlying the development of ocular manifestations remain unclear. For this reason, further studies are needed to elucidate the molecular pathogenesis of the development and progression of prematurely aging Werner syndrome.

## Figures and Tables

**Table 1:** Ophthalmological findings and procedures. VAs, visual acuities; OCT, optical coherence tomography; PEA + IOL, phacoemulsification, aspiration and intraocular lens implantation; CME, cystoid macular edema.

	OD (Right)	OS (Left)
VAs (before surgeries)	0.05 × S + 8.0D	0.7 × S + 3.75 Cyl-1.5D Ax 70
VAs (after surgeries)	1.2 × S + 0.5 Cyl-1.0D Ax 130	1.2 × S + 0.75 Cyl-1.0D Ax 60
Slit-lamp examinations	Milky and whitish cataracts	Cortical and posterior subcapsular cataracts
Axial lengths	21.6 mm	22.2 mm
Intraocular pressures (before surgeries)	19 mmHg	19 mmHg
Intraocular pressures (One day after surgeries)	34 mmHg	27 mmHg
Intraocular pressures under anti-glaucoma meds (Final visit)	18 mmHg	18 mmHg
Anterior OCT findings	Plateau iris	Plateau iris
Posterior OCT findings	Pachychoroid-like feature	Pachychoroid-like feature
Surgical procedures	PEA + IOL + goniosynechialysis	PEA + IOL
Surgical complications	Ocular hypertension Mild posterior subcapsular opacification No CME	Ocular hypertension Mild posterior subcapsular opacification No CME
Humphrey visual field tests	Normal	Normal

**Table 2:** Ocular manifestations in patients with Werner syndrome. In particular, pachychoroidal-like features and plateau iris are new findings in our case. OCT, optical coherence tomography; RNFL, retinal nerve fiber layer. Bold manifestations indicate that these findings were observed in our case.

Frequencies	Ocular Manifestations	References
Major	[**Bilateral cataracts**]	[1,2,11]
	The prevalence is over 90% in their 20s	[12]
	Subcapsular posterior cataracts	[20]
	Hypermature or Morgagnian cataracts	[21]
	Milky and opaque cataracts	[22]
	Nuclear sclerotic cataracts	[23]
Unknown	Glaucoma	[19,24,25]
Unknown	Spontaneous bullous keratopathy	[26]
	Corneal metastatic calcification	[27]
Unknown but thinning of the RNFL may be major	[Posterior OCT findings]	
	Thinning of the RNFL	[19]
	Thinning of the choroidal thickness	[19]
	**Pachychoroidal-like features**	**This case**
Unknown	[Anterior OCT findings]	
	**Plateau iris**	**This case**
Unknown	**Short axial lengths**	[21,22], **this case**
Rare	Retinitis pigmentosa-like features	[28]
Rare	Paramacular degeneration	[29]
Rare	Retinal detachment	[30]
Unknown but probably rare after small incision surgeries	[Surgical complications]	
	Wound dehiscence-related complications	[20,31,32]
	Corneal endothelial decompensation	[20,31]
	Cystoid macular edema	[23,33,34]
	**Ocular hypertension**	**This case**
	**Posterior subcapsular opacification**	[23,35], **this case**

## Data Availability

The original contributions of this study are presented. Further inquiries can be directed to the corresponding author.
